# Non-*Mastomys* rodents harbour ancient Lassa virus lineages within Benin and Nigeria’s Guinea savanna belt

**DOI:** 10.1038/s41598-026-51525-8

**Published:** 2026-05-09

**Authors:** Ayodeji Olayemi, Adetunji Samuel Adesina, Akinlabi Oyeyiola, Adeoba Obadare, Umaru Bangura, Nnennaya Anthony Ajayi, Kingsley Ukwaja, Liman Mohammed, Adamu Ibrahim, Toni Rieger, Stephan Günther, Anges Yadouleton, Elisabeth Fichet-Calvet

**Affiliations:** 1 Natural History Museum, Obafemi Awolowo University, Ile Ife, Osun Nigeria; 2 Zoonoses Control Research Group, Bernhard Nocht Institute for Tropical Medicine, Hamburg, Germany; 3 Department of Biochemistry and Molecular Biology, Obafemi Awolowo University, Ile Ife, Osun Nigeria; 4 Federal Teaching Hospital Abakaliki, Abakaliki, Ebonyi Nigeria; 5 Ministry of Health, Lafia, Nasarawa Nigeria; 6 Virology Department, Bernhard Nocht Institute for Tropical Medicine, Hamburg, Germany; 7 Laboratoire des Fièvres Hémorragiques Virales, Cotonou, Benin

**Keywords:** Ecological zonation, Lassa virus reservoir, Multimammate mice, non-*Mastomys* rodents, Novel lineage, Savanna, Ecology, Ecology, Microbiology, Zoology

## Abstract

The zoonotic Lassa virus (LASV), killing thousands of people annually, is maintained majorly by the multimammate mice *Mastomys natalensis* and *M. erythroleucus*; but other rodent reservoirs exist. Knowledge regarding LASV ecology remains limited in certain areas where Lassa fever continues to be incident and even recently emergent. Here, we assess community composition and LASV infection-status of small mammals in key localities across the Guinea savanna belt that spans the countries of Benin and Nigeria. Active LASV infection was determined by conventional (gel based)- and quantitative-PCR screening, while previous infection was determined by an immunofluorescent assay on IgG antibodies. Surprisingly, *M*. *natalensis* and *M. erythroleucus*, known to be sympatric across the Guinea savanna, co-occurred in only one locality and were PCR-negative for the virus; but showed IgG antibodies in localities endemic for Lassa fever within Nigeria. Viremia, alternately, was detected in *Mus* (*Nannomys*) *baoulei* and (a newly-discovered reservoir) *Lemniscomys striatus*, carrying relatively ancient LASV lineages, VIII & IX respectively, within Benin. Our results suggest non-*Mastomys* rodents assume an increased role in LASV ecology within the Guinea savanna belt of Benin and Nigeria; especially in central Benin, which turns out to be a long-overlooked hotbed of early evolution, host-switching and contemporary emergence.

**Supplementary Information:**

The online version contains supplementary material available at 10.1038/s41598-026-51525-8.

## Introduction

Lassa fever, a deadly zoonotic disease, is estimated by current models to have an incidence of up to 879,700 and to kill up to 18,000 people across West Africa every year^1^. It commonly involves febrile symptoms which may advance to haemorrhaging and result in death^2^. Rodents are the natural reservoir of the causative *Mammarenavirus lassaense* (Lassa virus -LASV), and humans become infected when they come in contact with droppings of infected rodents^3^. Five rodent species have currently been confirmed as natural LASV reservoirs across West Africa: the Natal multimammate mouse *Mastomys natalensis* in all countries within West Africa endemic for Lassa fever^3–7^, the Guinea multimammate mouse *M. erythroleucus* in Guinea, Nigeria and Sierra Leone^8,9^, the African wood mouse *Hylomyscus pamfi* in Nigeria^8^, the Pygmy mouse *Mus baoulei* in Ghana and Benin^10,11^, and the Rusty-bellied rat *Lophuromys sikapusi* in Sierra Leone^9^. Active LASV infections have been repeatedly detected by PCR or isolation in taxonomically-verified individuals of each of these rodent species^12^. Among these reservoirs, the *Mastomys* species have been shown to possess greater zoonotic capacity in terms of their LASV prevalence^4,5,7,13–15^, their relative abundance^16^ and their commensal life history^12,14,17,18^.

**Fig. 1:**
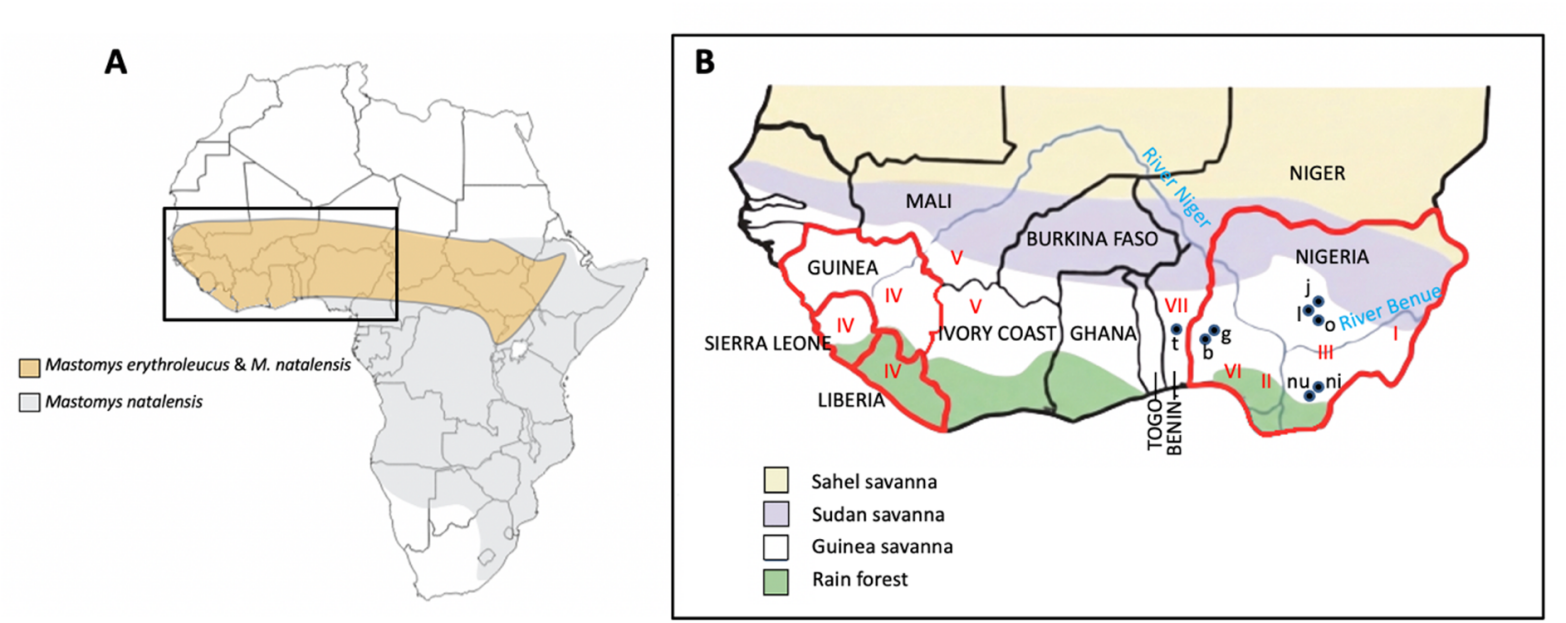
Sampling area; situated in context of the occurrence of major known LASV-reservoirs, -lineages, -endemic areas as well as ecological zones. (**A**) Map of Africa showing distribution of the main LASV rodent reservoirs, *Mastomys natalensis* and *M. erythroleucus*, following^23^. These two *Mastomys* species overlap across western and central Africa, particularly in the savannas. The black box encloses western Africa, the broad region of Lassa fever occurrence within the continent. (**B**) Map of West Africa showing countries commonly regarded as endemic for Lassa fever (with red borders), LASV lineages detected in rodents, indicated in roman numerals (except I, the prototypic lineage, described from the first humans described with Lassa fever), and ecological zones. Black dots represent localities sampled in this study within the Guinea savanna of Benin and Nigeria for small mammals: “t”, the commune of Tchaourou, comprising Gango, Kadjola, Kassouala, Odo-akaba, Worogui & Tambouan; “b”, Bwen; “g”, Gwanara; “l”, Lafia; “j”, Jos; “o”, Obi; “nu”, Ndubia; “ni”, Ndiawala.

With regard to frequency of epidemics and prevalence of the virus, two endemic areas are recognised for Lassa Fever within West Africa: Nigeria on one hand and the Mano River Union Countries (MRU, comprising Guinea, Liberia & Sierra Leone) on the other (Fig. 1)^19^. Surveys to evaluate various aspects of the zoonotic risk presented by small mammals; i.e., their community composition, level of commensality, accurate identification of reservoir species, lineage classification of detected virus variants, and serological surveillance to describe the extent of previous, cleared infections, have been mostly focused in these LASV-endemic areas^4,7,9,14,16,20,21^. Outside the endemic areas, preliminary small mammal surveys for LASV have been carried out within Benin^10^, Ghana^11^, Ivory Coast^6,22^ and Mali^5^, where outbreaks are sporadic.

Benin has long been regarded as a non-endemic country for Lassa fever, but then epidemics were recorded during 2014 and 2016; with speculations that cross-border transmission between Nigeria and Benin may have been involved^24,25^. To search for LASV reservoirs, six villages were sampled for small mammals during September 2017 in central Benin, the area of the 2016 epidemic. Complementary trapping was carried out during the same month in two villages across the border within Nigeria. A gel-based PCR revealed active infections in three Pygmy mice of the species *M.* (*Nannomys*) *baoulei*, representing novel LASV variants close to lineage VII within Benin^10^. This demonstrates that more needs to be understood regarding the role that small mammals play in LASV evolution and epidemiology within this region.

Nigeria, adjacent to Benin, is endemic for Lassa fever. However, though human cases occur over significant portions of Nigeria, most studies on small mammals are focussed in the southern part of the country where the rainforest occurs^7,8,15,16,18,20^. Thus, localities where Lassa fever recently emerged during 2016 in central Benin^24^ and those to the immediate north of the rainforest within Nigeria, which records continual Lassa Fever incidence (Table 1) - are in need of more comprehensive small mammal surveys for LASV.

**Table 1:** Laboratory-confirmed cases of Lassa fever documented by the Nigeria Centre for Disease Control^26^ within Ebonyi, Kwara, Nasarawa and Plateau, states containing the Nigerian localities sampled for small mammals in this study. This compilation represents human cases diagnosed during 2017, the year small mammal trapping was conducted for almost all the sites in our survey.

	January	February	March	April	May	June	July	August	September	October	November	December
Ebonyi		2						2				
Kwara												
Nasarawa	2		1	1								1
Plateau		3					8	2			1	

Worthy to note is that these localities are contained within the Guinea savanna belt, which runs through both countries (Fig. 1). Like in Benin, clearer insight is needed within the Guinea savanna in Nigeria concerning the specific small mammal reservoirs of LASV. An early key investigation involved isolation of LASV from rodents; reportedly of the species *M. natalensis*,* Rattus rattus* and *Mus minutoides* in the locality of Jos and its environs around central Nigeria^27^. However, the authors of this finding mentioned that “with difficulties inherent in rodent classification under austere field conditions, the possibility that misidentifications may have occurred must be strongly considered”. Indeed, due to similarity in external morphology, small mammals from the genera *Mastomys* and *Mus*, for instance, can be notoriously subject to erroneous designation^28–30^. To avoid this pitfall, contemporary studies in LASV ecology increasingly incorporate tools such as molecular taxonomy to reliably identify small mammals^12^.

Another glaring knowledge-gap concerns the potential for elevated, joint amplification of LASV between *M. natalensis* and *M. erythroleucus* within the Guinea savanna, where these multimammate mice are sympatric. *M. erythroleucus* occurs in the savanna belt and degraded rain forests that extend across West- and Central Africa. The distribution of *M. natalensis* overlaps with *M. erythroleucus* in this region, as *M. natalensis* is present throughout most of sub-Saharan Africa (Fig. 1)^23^. LASV transmission was recently described between these two taxa in a fragmented-forest site within south western Nigeria where LASV lineage II circulates (Fig. 1)^20^. Infection of both *M. natalensis* and *M. erythroleucus* with LASV lineage II within the rainforest demonstrates there is heightened potential for the virus to spread to populations of either of these species that are still naïve across Nigeria; especially in the Guinea savanna, which possibly has a higher number of localities where *M. natalensis* and *M. erythroleucus* co-occur.

With regard to differences in the proportion of virus-positive individuals between reservoir populations, LASV lineage II, with prevalence reaching up to 76%, has been found for *M. natalensis* in surveys conducted across the Edo-Ondo hotspot in the rainforest zone of southwestern Nigeria^20^. LASV-positive *M. erythroleucus* in the same locality where *M. natalensis* carrying the virus were also captured, recorded a prevalence of 16.18% (11/68 individuals). However, in one of the few preliminary rodent surveys documented within the Guinea savanna, only three PCR-positive individuals (carrying LASV lineage III) were realised out of 63 *M. erythroleucus* trapped on the southern bank of the Benue River^8^. It has not been assessed if this pattern continues into central Nigeria and to what extent *M. natalensis* (which was also present on the southern bank of the Benue River but LASV-negative) may serve as a joint reservoir of this particular lineage. Furthermore, IgG antibodies (but not LASV viremia) were detected in *M. erythroleucus* within Ndubia, where the Guinea savanna extends into the southeastern part of Nigeria^15^ (Fig. 1). Thus, from results obtained so far, the general pattern appears to be that *Mastomys* populations within the rainforest zone are considerably more prevalent with LASV than those in the savanna.

In this study, we sought to describe small mammal community and commensality in certain localities within central Benin where Lassa Fever emerged recently, but also in the Guinea savanna within Nigeria where incidence occurs yet knowledge-gaps exist regarding the ecology of this disease. For LASV, we assessed virus- and sero-prevalence and phylogenetically classified variants we detected. Furthermore, to gauge the impact of ecological zonation, we compared findings from our survey – which was carried out in the Guinea savanna – to previous reports from the rainforest.

## Materials and methods

The Guinea savanna in western Africa, broadly defined, is the biotic zone which lies along the immediate north of the rainforest belt^31,32^ (Fig. 1). This ecological zone, which extends southward down to the coast in the Republic of Benin^33^, is characterised by grassland interspersed with trees; typical woody species include *Daniella oliveri*, *Afzelia africana*, *Isoberlinia doka* and *Monotes kerstingii*^34^. Compared to the rainforest, the Guinea savanna records more variable seasonal conditions annually: between 1000 and 1500 mm in total rainfall and 20 and 33 °C in mean temperature^35^.

### Study sites

A total of 13 localities were sampled for small mammals in this study across the Guinea savanna belt that is continuous between Benin and Nigeria (Fig. 1).

- Following the 2016 Lassa fever epidemic in Benin^24^, we sampled six villages in the commune of Tchaourou, department of Borgou, during September 2017: Gango (8° 58′ N, 2° 41′ E), Kadjola (8° 54′ N, 2° 43′ E), Kassouala (8° 52′ N, 2° 45′ E), Odo-akaba (8° 46′ N, 2° 36′ E), Worogui (8° 53′ N, 2° 40′ E) and Yambouan (8° 58′ N, 2° 45′ E). Two localities in Kwara State, Bwen (8° 50′ N, 2° 53′ E) and Gwanara (8° 54′ N, 3° 07′ E), were also sampled in Nigeria along the border in the same month.
- During November 2017, our sampling was extended into three localities within central Nigeria: Jos, Plateau State (9° 51′ N, 8° 58′ E), Lafia, Nasarawa State (8° 30′ N, 8° 30′ E) and Obi, Nasarawa State (8° 22′ N, 8° 46′ E), where Lassa fever is known to be endemic, occurring annually^26^.
- In July 2019 we also sampled Ndubia (6° 21′ N, 8° 19′ E) and Ndiawala (6° 40′ N, 8° 11′ E) within Ebonyi State, where the Guinea savanna extends into southeastern Nigeria. This area is also endemic for Lassa fever^26^, with Ndubia being a locality where our team had preliminarily sampled small mammals for LASV in 2015^15^. The 2015 survey detected only seropositive, but not actively infected, *M. erythroleucus* rodents.

All these sites occur at less than 500 m above sea level (abs); except the Jos Plateau, which is as high as 1200 m abs^34^.

### Trapping and small mammal identification

Small mammals were captured using Sherman live traps (7.5 by 9 by 23 cm, www.shermantraps.com) baited with a paste of peanut butter, flour and fried fish. Traps were set indoors in a transect through the localities that were smallest in size (i.e., all the villages within Benin; and Bwen, Gwanara and Ndiawala within Nigeria). A minimum of four traps were set in each building, with two traps despatched per room in living and food storage areas, bedrooms and kitchens. Additionally, four trap-lines each comprising 20 traps (with five-metre spacing between the traps) were erected outdoors in each of these villages along wild vegetation and in peri-domestic spots such as gardens and trash heaps.

In the larger localities (Jos, Lafia, Obi and Ndubia), traps were set at addresses dotting the sampling area. Trapping was carried out both within and outside houses in Jos and Ndubia. In Jos, trapping was conducted in Amurum village, beside the A.P. Leventis Ornithological Institute. In Ndubia, houses surrounding St Vincent’s Hospital were sampled. In Lafia and Obi, for logistic reasons, trapping could only be done indoors. Here, sampling addresses included some that had recorded a confirmed case of Lassa fever within recent years. The trapping effort for each locality in this study, expressed in “trap-nights” (a total, combining the number of traps set every night through the duration of trapping) appears in the Table 2. Overall, a total effort of 6604 trap-nights was expended. Small mammals were identified by external morphology using (Happold 2013) as a guide. Due to phenotypic similarity, congeneric specimens within *Mastomys* and *Mus* were identified by Cytochrome *b* DNA sequencing^36^.

### Lassa virus testing

LASV infections were tested by conventional, endpoint PCRs to detect the glycoprotein complex (GPC-specific to LASV^37^), and L-fragment (targeting a broad range of mammarenaviruses^38^), in RNA extracted from whole-blood samples. Results from the endpoint PCR screening have been published for the six villages from Benin^10^. As our earlier surveys into the Guinea savanna yielded low prevalence^8,15^, and, in order to reduce the possibility of false-negatives, we carried out additional, quantitative screening (qPCR, which carries a higher sensitivity^39^) for all the localities in Benin and also for Lafia, Ndiawala and Ndubia within Nigeria. In our qPCR, RNA extracted from small mammal blood samples were tested using the RealStar^®^ Lassa Virus RT-PCR Kit 2.0 (Altona Inc. Germany), targeting the L and the GPC genes on a Rotor-Gene^®^6000 (Corbett Research) thermal cycler. For each run, only samples with a cycle threshold (Ct) value below that of the positive control were regarded as LASV-positive. GPC 1 kb fragments were Sanger-sequenced from LASV-positive animals using the following primers: Forward:

OWS0001: 5′-GCGCACCGGGGATCCTAGGC-3′ and Reverse: OWS 1000: 5′-AGCATGTCACAGAAYTCYTCATCATG-3′^40^. Forward and reverse sequences were assembled in the software MacVector 18.8.2 (MacVector Inc., USA) and then aligned with other published sequences.

### Phylogenetic analysis

In total, 50 partial LASV sequences of GPC (910 nt) were aligned, including variants from the 7 lineages to ensure reliable positioning of the new variants. In BEAUTI, the parameters were as follows: the tip dates at the nearest day; a substitution model as GTR + gamma and codon partition with positions 1, 2, 3; a strict clock; a coalescent tree with a constant size population; and a MCMC = 10 M, echo states, and log parameters every 10,000. The xml files issued from BEAUTI were run in BEAST, the log files checked in TRACER, and consensus trees were visualized through FigTree (BEAST packages, https://beast.community/programs)^41^.

### Serology

We also tested our small mammal blood samples from all 13 localities for indications of previous LASV infection using an Immunofluorescence Assay (IFA) for IgG antibodies. As in^42^, microscope slides bearing cells infected with the Bantou LASV strain (from lineage IV of the virus) were prepared in the Biosafety level 4 Laboratory at the Bernhard Nocht Institute for Tropical Medicine, Hamburg, Germany. The cells were permeabilized and the complex viral nucleoprotein-IgG clearly appeared as yellow dots in the cells under the microscope with UV light. The reaction was validated in^43^ and we used an anti-LASV nucleoprotein monoclonal antibody, developed by^44^, as positive control. Seven microlitres of a solution containing small mammal blood in Phosphate Buffered Saline (PBS, 1:20) was pipetted into each slide well and incubated at 37 °C. Anti-mouse Immunoglobin G (IgG) Fluorescein Isothiocyanate (FITC) was added and the slides viewed under a fluorescence microscope after a second incubation. For each of our 12-well slides, one well (spotted with mere PBS) represented a negative control and another represented the positive control.

Evidence is convincing that our assay, employing the Bantou strain as antigen, is satisfactorily sensitive to detection of IgG antibodies generated by other LASV lineages and mammarenaviruses. Our positive control never failed in all experiments and previous small mammal surveys within Nigeria using this IFA have yielded significant seroprevalence in localities where LASV lineages II, III, VI and a Mobala-like mammarenavirus circulate^15,16^. Moreover, a recently-validated enzyme-linked immunosorbent assay (ELISA), which employs the LASV AV (lineage V) strain as antigen, showed 97.1% concordance when compared with IgG seroprevalence obtained from Bantou antigen-based IFA screening of the same small mammal collection gathered across various West African countries^45^.

Because LASV antibodies are known to cross-react with other mammarenaviruses, the identity of viruses inducing IgG signals in our small mammals was inferred by (a) the presence of other conspecific individuals which were virus-positive in the same locality, and (b) by the epidemiological history of IgG-positive small mammals in question—in the absence of virus-positive individuals in the vicinity.

## Results

### Small mammal distribution

**Fig. 2:**
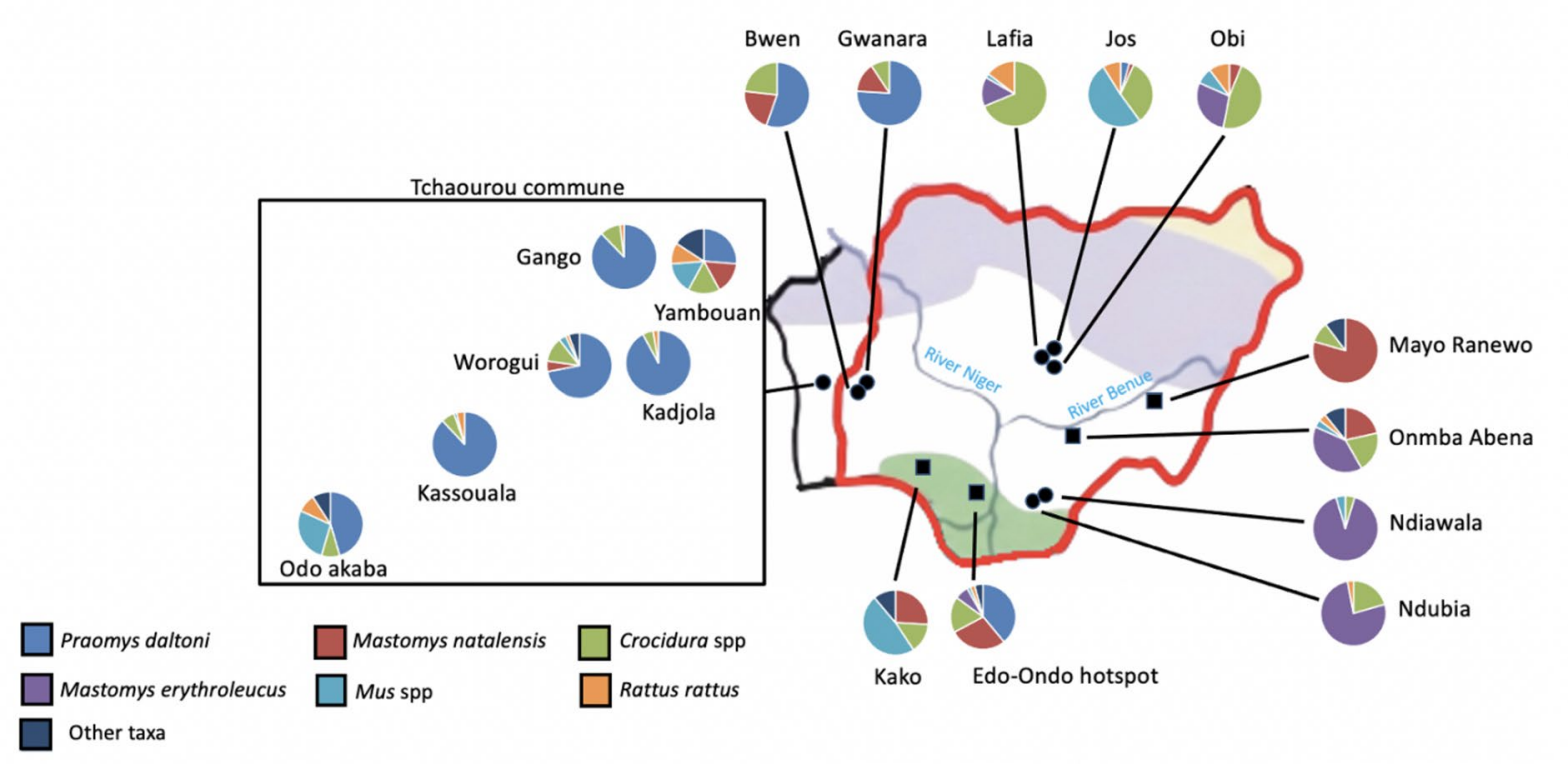
Small mammal composition per locality. Black circular dots indicate sites sampled within the Guinea savanna. Pie charts portray proportions of respective taxa, with actual capture numbers listed in Table 2. For comparison, relevant small mammal communities from previous studies where specimens were reliably identified are included, indicated by black square dots. These include Kako (*n* = 27^18^), Onmba Abena (*n* = 158^18^), Mayo Ranewo (*n* = 39^18^), and the Edo-Ondo hotspot (*n* = 1116^16^).

A total of 614 small mammals were captured, comprising at least 11 species (Table 2; Fig. 2). The rodent *Praomys daltoni* was the most abundant, with 284 individuals collected. *Crocidura* shrews were the second most numerous, consisting of 124 individuals. The multimammate mice followed, featuring 86 *M. erythroleucus* and 30 *M. natalensis*. These *Mastomys* species, both known as major LASV reservoirs, co-occurred only in one locality: Obi. Additionally, regarding geographic distribution, *Crocidura* spp., *M. natalensis*, *P. daltoni* and *R. rattus* were relatively widespread across the study area; compared to other taxa which were encountered on only one side of the Niger River, a prominent topographic barrier. *Lemniscomys striatus*, *M.* (*N*.) *baoulei*, and *Mus* (*N*.) *mattheyi* were captured only to the west of the Niger River; and *M. erythroleucus*, *M. musculus*, *Mus* (*N*.) *musculoides* and *Mus* (*N*.) *minutoides* to the east (Table 2; Fig. 2). Majority of the small mammals were trapped indoors (510 out of 614). Even disregarding results from Lafia and Obi, where trapping could not be carried out outside, every locality had more individuals captured inside than in outdoor habitats (Table 2). Taxon-wise, the more abundant small mammals recorded higher captures indoors. The opposite was the case for those taxa that had less than 10 individuals trapped; i.e., the Striped grass mouse *L. striatus* and the Pygmy mice (*Nannomys*) (except *M.* (*N*.) *musculoides*).

**Table 2:** Small mammal distribution and infection status across the study area. For each small mammal taxon, the number of individuals captured per locality appears on the upper row; beside which the number that tested positive appear in parenthesis (PCR+/IgG+). The lower row against each taxon expresses individuals captured indoors/those trapped outdoors. Lafia and Obi were trapped only indoors; outdoor data in these localities are thereby denoted “–”.

Sampling date	Worogui, Tchaourou	Kassouala, Tchaourou	Kadjola, Tchaourou	Yambouan, Tchaourou	Gango, Tchaourou	Odo-Akaba, Tchaourou	Bwen, Kwara State	Gwanara, Kwara State	Jos, Plateau State	Lafia, Nasarawa State	Obi, Nasarawa State	Ndubia, Ebonyi State	Ndiawala, Ebonyi State	Total
	Sept 2017								Nov 2017	Nov 2017	Nov 2017	July 2019	July 2019	
Trapping effort	600	600	600	600	600	600	526	450	470	365	431	365	397	6604
*Crocidura spp*	7	5	2	3	5	4	13	5	15(0/1)	33	23	7	2	124(0/1)
	1/6	1/4	0/2	0/3	1/4	1/3	3/10	2/3	9/6	33/-	23/-	7/0	2/0	83/41
*Lemniscomys striatus*	3(1/0)			3(2/0)		4(0/1)								10(3/1)
	0/3			0/3		0/4								0/10
*Mastomys erythroleucus*										7(0/1)	14	26(0/4)	39	86(0/5)
										7/-	14/-	26/0	39/0	65/0
*Mastomys natalensis*	3			3			12	8	1		3			30
	0/3			3/0			12/0	7/1	0/1		3/-			25/5
*Mus* (*Mus*) *musculus*									21(0/4)	1				22(0/4)
									19/2	1/-				20/2
*Mus* (*Nannomys*) *baoulei*	2(2/0)					12(1/5)								14(3/5)
	0/2					0/12								0/14
*Mus* (*Nannomys*) *mattheyi*	3	1		3										7
	1/2	0/1		2/1										3/4
*Mus* (*Nannomys*) *musculoides*											4		2	6
											4/-		0/2	4/2
*Mus* (*Nannomys*) *minutoides*									2					2
									0/2					0/2
*Praomys daltoni*	41	66	35(0/1)	5	43	20	31	41	2					284(0/1)
	41/0	62/4	29/6	3/2	41/2	20/0	30/1	34/7	2/0					262/22
*Rattus rattus*	1	3	1	2	1	4			4	7	5	1		29
	0/1	3/0	1/0	2/0	0/1	4/0			4/0	7/-	5/-	1/0		27/2
Total	60	75	38	19	49	44	56	54	45	48	49	35	43	614 (6/17)
	43/17	66/9	30/8	10/9	42/7	25/19	45/11	43/11	34/11	48/-	49/-	35/0	41/2	510/104

### LASV infection status

**Fig. 3:**
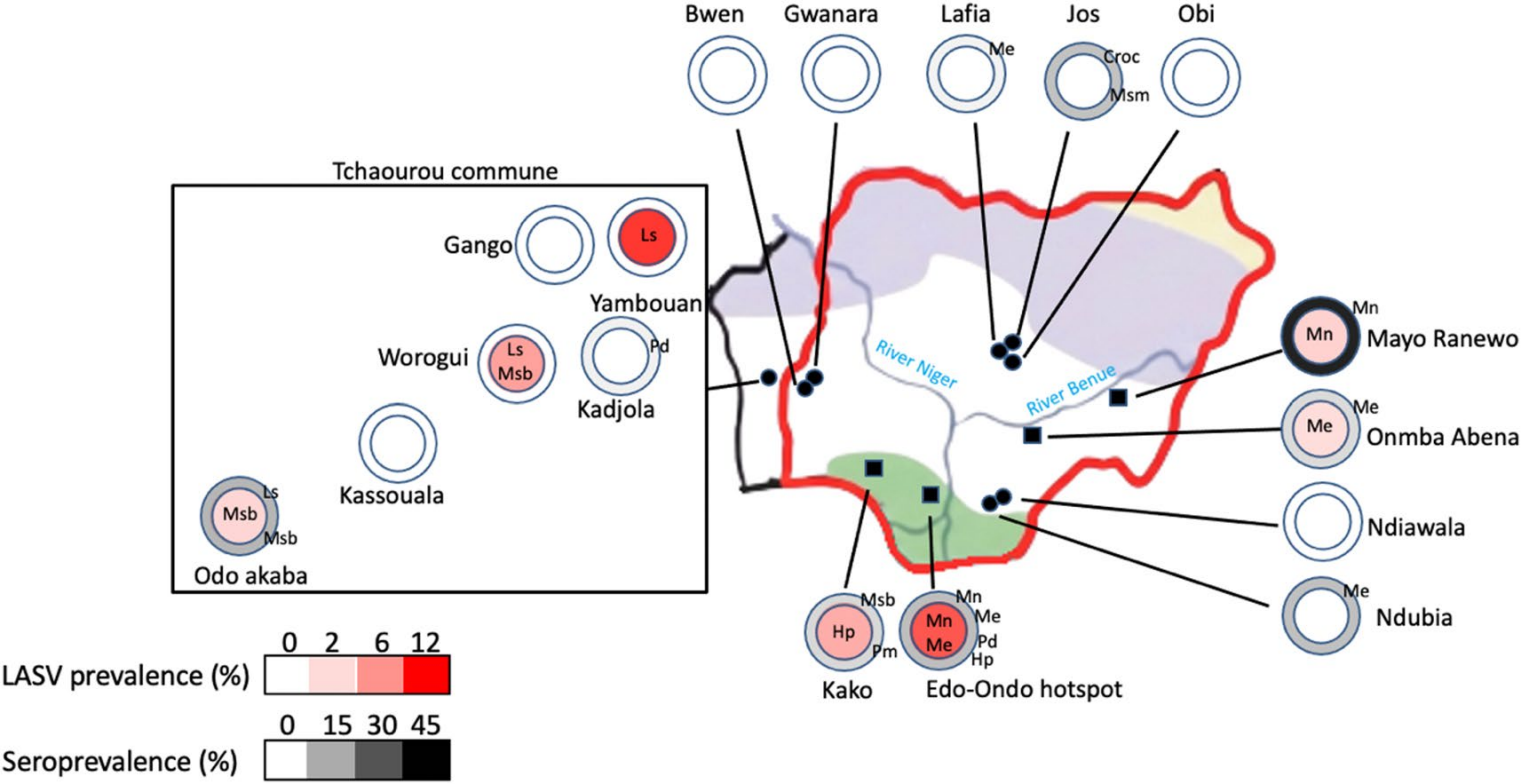
Virus- and sero-prevalence, based on the total number of small mammals captured in each locality. Black circular dots indicate sites sampled within the Guinea savanna. Actual capture numbers are listed in Table 2. For comparison, prevalence values from relevant previous studies where specimens were reliably identified are included, indicated by black square dots. These include Kako (*n* = 27^18)^, Onmba Abena (*n* = 158^18)^, Mayo Ranewo (*n* = 39^18^), and the Edo-Ondo hotspot (*n* = 1116^16^). The inner portion of the larger cycles represent LASV prevalence (except in Mayo Ranewo, where a Mobala-like mammarenavirus was detected in a previous study). Outer portions of the larger circles portray seroprevalence. Abbreviations inside circles represent small mammal taxa that were virus-positive, while taxa on the outside represent those seropositive: Croc, *Crocidura* spp; Hp, *Hylomyscus pamfi*; Ls, *Lemniscomys striatus*; Me, *Mastomys erythroleucus*; Mn, *Mastomys natalensis*; Msb, *Mus* (*Nannomys*) *baoulei*; Msm, *Mus musculus*; Pd, *Praomys daltoni*.

**Fig. 4:**
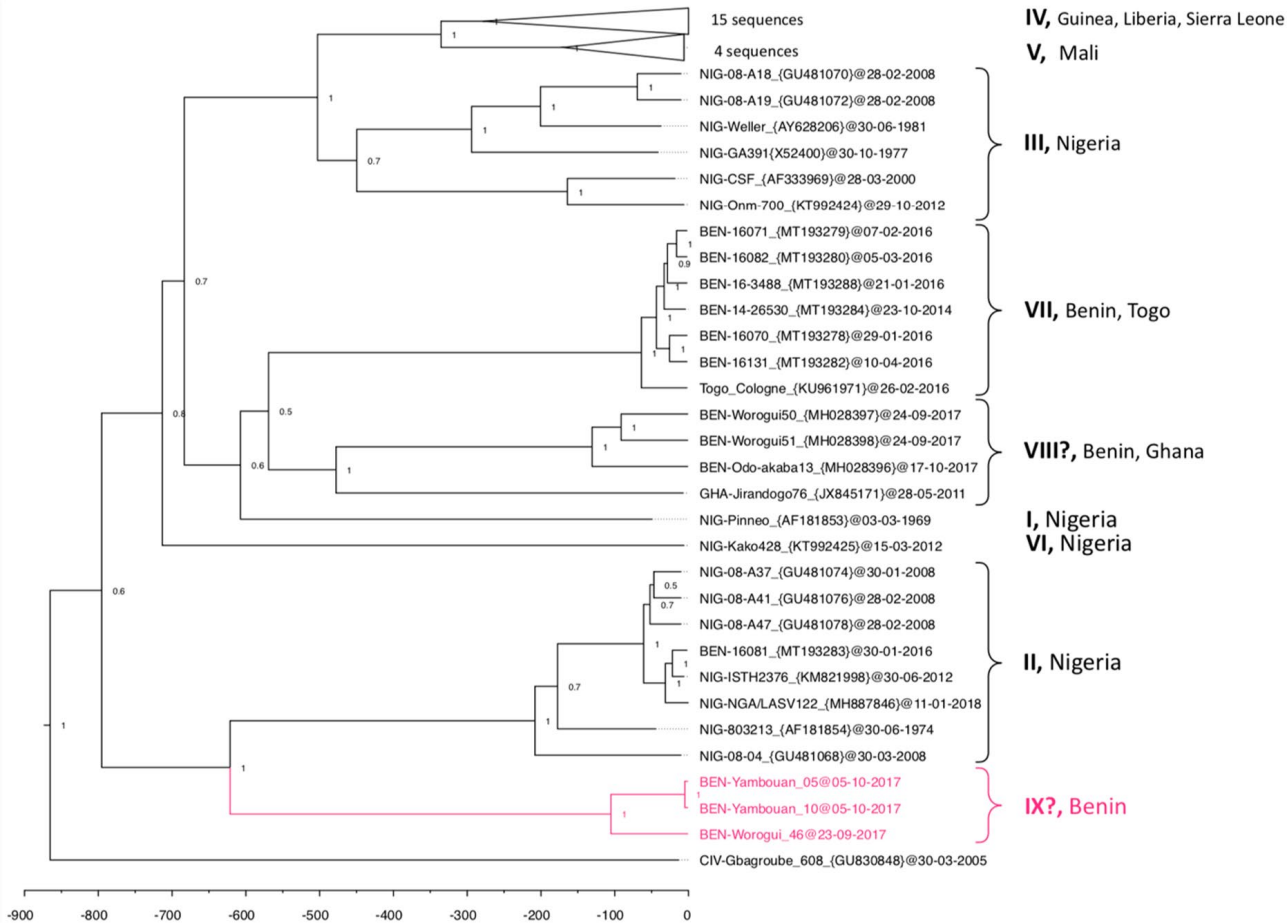
Phylogenetic tree of Lassa virus based on partial GPC sequences (910 nt). Newly identified variants from *Lemniscomys striatus* in Benin are highlighted in red. LASV sequences are available in GenBank under accession numbers PX994346-48.

Screening all small mammals in this study by gel-based PCR revealed active LASV infections in Baoule’s pygmy mouse *M.* (*N*.) *baoulei* in Benin: 2/2 individuals in Worogui (100%) and 1/12 (8.33%) in Odo-Akaba. Further testing by qPCR also detected active infections in the Striped grass mouse *L*. *striatus*: 1/3 individuals (33%) in Worogui and 2/3 (66%) in Yambouan (Table 2; Fig. 3, Supplementary Fig. S1). For the three LASV-positive *L. striatus*, genetic sequences were submitted to GenBank (LASV accession numbers PX994346-48, Supplementary Text 1; rodent Cytochrome *b* accession numbers PX994349-351, Supplementary Text 2). Phylogenetic analysis assigned the LASV sequences from *M.* (*N*.) *baoulei* as lineage VIII, while the novel variants from *L. striatus* formed a distinct but sister-clade to lineage II, designated here as lineage IX (Fig. 4). This lineage is strongly supported (posterior value = 1) and diverged from lineage II approximately 600 years ago. Given its basal position and proximity to lineage II, *L. striatus* is unlikely to be the reservoir for LASV strains (lineage VII) circulating among humans in the same region. These results establish that both LASV lineages VIII & IX - borne by non-*Mastomys* reservoirs, *M.* (*N*.) *baoulei* & *L*. *striatus* respectively – circulate around central Benin and particularly co-occur in the locality Worogui.

The rodent species detected to be PCR-positive for LASV in this study also had individuals that were IgG-positive in Odo-Akaba: 5/12 (41.7%) for *M.* (*N*.) *baoulei* and 1/4 (25%) for *L. striatus* (Fig. 3). Small mammal taxa which were not found to carry active LASV infections but tested seropositive were present in the study area. *M. erythroleucus* was IgG-positive in Lafia (seroprevalence 1/7, 14.29%) and Ndubia (4/26, 15.38%), while *P*. *daltoni* was IgG-positive in Kadjola (1/35, 2.86%). Other small mammals were seropositive in Jos: *Crocidura* spp (1/15, 6.67%) and *M. musculus* (4/21, 19.06%).

## Discussion

### Small mammal commensality, community composition and co-occurrence of *Mastomys* species

83% (510/614) of the small mammal individuals in our study were captured indoors, highlighting the extent of commensality represented by our collection. Indeed, taxa that were the most abundant such as *Crocidura* spp. and *P. daltoni*, or at least among the most widely distributed (e.g., *M. natalensis* and *R. rattus*), are well known commensals. Regarding taxonomic diversity, recent surveys recorded at least nine species each in Benin^46^ and in localities reaching into the savanna zone within Nigeria^18^. These results are comparable to the eleven taxa realised in this particular survey. It is also important to note that certain species are not common to both countries (such as *Nannomys musculoides*, which has not been detected in Benin, and *Nannomys mattheyi*, yet un-trapped in Nigeria). Our study concurred with the known distribution of several taxa, including most of the *Nannomys*. In agreement with previous studies, *N. mattheyi* occurs in Benin but is not distributed into Nigeria, *N. baoulei* occurs in both Benin and Nigeria but only to the west of the Niger river, while *N. musculoides* occurs in Nigeria but only to the east of the Niger river^10,18^.

Distribution-wise, other taxa were underrepresented in our collection. We captured *L. striatus*, *M. erythroleucus* and *N. minutoides* only on one side of the Niger river in our study, but these species have been confirmed in prior surveys to occur on both sides^10,18,20^. Conversely, we add new knowledge concerning the geographic distribution of *P*. *daltoni*, which we detected in Jos, to the east of the Niger river, and *M. musculus*, which we trapped in Jos and Lafia, in central Nigeria. Up till now, these species have been known to be restricted to the western side of the Niger river and to southern coastline localities within Nigeria, respectively^18,23,47^.

Part of our motivation for this investigation was to explore the potential for synergistic LASV-proliferation between *M. natalensis* and *M. erythroleucus* in the savanna, as had been recently detected in the Edo-Ondo hotspot within the rainforest zone of southwestern Nigeria. However, we found co-occurrence between *M. natalensis* and *M. erythroleucus* to be limited; recorded only at Obi. Revisiting earlier studies, it appears this may be a more common pattern: In Guinea, *M. erythroleucus* was trapped more in coastal villages, while *M. natalensis* was more dominant inland^4,48^. In a transect running through the countries of Benin northward into the Republic of Niger, *M. natalensis* was captured at almost every site, while *M. erythroleucus* was detected only in the northernmost site within the Republic of Niger^46^. More recently, in Senegal, where *M. erythroleucus* was recorded in 33 localities and *M. natalensis* 6, both species co-occurred in only 3 localities^49^. Thus, though *M. natalensis* and *M. erythroleucus* are described as sympatric across the savanna zone of West Africa^23^, surveys increasingly demonstrate that, on a finer scale (i.e., at the local and habitat level), the existence of both species is not totally concordant.

### LASV/mammarenavirus circulation per small mammal species

#### Active infection

Most mammarenavirus infections in small mammals are acute^42,50–52^, and gauging the prevalence of active LASV infection helps inform the potential for spread between animal reservoirs and spillover to humans. Our study recorded LASV RNA in only 6/614 individuals and 2/13 localities of the savanna belt spanning Benin and Nigeria. *Mastomys* rodents are the preeminent LASV reservoirs, yet remarkably, no individuals from this genus were captured with LASV viremia, even in localities known to be endemic for Lassa fever; though several *Mastomys* showed IgG antibodies, signalling cleared infection. This supports the idea that ecological zonation may affect LASV maintenance in small mammals, with prevalence in the savanna belt of Nigeria and Benin being markedly lower compared to that of rainforest localities in southern Nigeria, where a PCR-prevalence of up to 76% has been recorded^20^.

Indeed, even though IgG antibodies have been detected in *Mastomys* within the Guinea savanna of Nigeria, so far, to the best of our knowledge, only three taxonomically-verified *M. erythroleucus* individuals have been detected with active LASV infection^8,15^. By the same measure, no taxonomically-verified *M. natalensis* has thus far been detected with an active LASV infection; though a Mobala-like mammarenavirus was found in a single *M. natalensis* in Mayo Ranewo (Fig. 3)^8,15^. This savanna-forest disparity in LASV prevalence between *Mastomys* populations within Nigeria seems to be reflected in the incidence of Lassa fever among humans. Through 2017–2019, the period our sampling was conducted, a total of 700 Lassa fever cases were confirmed in Edo State alone within the rainforest, compared to 131 and 28 in Ebonyi and Nasarawa states respectively in the Guinea savanna^53^. Rainfall, and to a lesser extent, temperature, have been proffered as leading environmental variables that might influence LASV maintenance in nature (and, by implication, its rodent reservoirs)^54^. These, and other possible intrinsic drivers such as neutral- and immuno-genetics^50^ of rodent-reservoirs should be investigated further to increase insight into variation in rodent-borne LASV prevalence between ecological zones.

LASV viremia in our study was detected in non-*Mastomys* rodents within Benin: 3 individuals each of Baoule’s pygmy mouse *M.* (*Nannomys*) *baoulei* (preliminarily reported in^10^) and the Striped grass mouse *L. striatus*. The Striped grass mouse—captured with virus RNA in Worogui and Yambouan, separate localities within central Benin – is identified in this study as an additional LASV reservoir and represents a contribution to what has been described as a niche of increasing importance: the evolution and emergence of LASV lineages hosted by non-*Mastomys* rodent reservoirs in an area situated broadly between the two major endemic zones for Lassa fever within western Africa^12^. Apart from *L. sikapusi* in Sierra Leone, this is the case for each of the taxonomically-verified non-*Mastomys* reservoirs that have been described so far: *L. striatus* in Benin (this study), *M.* (*Nannomys*) *baoulei* in Benin and Ghana^10,11^ and *H. pamfi* in a locality within southwestern Nigeria west of the Edo-Ondo hotspot^8^ (Fig. 3). *H. pamfi* and *M.* (*Nannomys*) *baoulei* appear to have been involved in the early evolution of LASV, as they host lineages VI & VIII respectively; which, like lineage I, are basal to most other lineages of the virus (Fig. 4). These ancient LASV lineages display varying levels of epidemiological importance. Lineage I was isolated from among the first humans diagnosed with Lassa fever during 1969 in north-eastern Nigeria^55^, but seems to have become extinct. No cases have been reported in humans within Kako, the locality where lineage VI was discovered in *H. pamfi*, while lineage VII was only recently detected in humans in Benin and Togo from 2016^10,56^.

LASV-positive *L. striatus* in this study, like the *M.* (*Nannomys*) *baoulei* and *H*. *pamfi* reservoirs, are found in localities where overlapping *Mastomys* populations have tested LASV-negative. However, *L*. *striatus* hosts lineage LASV IX, a separate yet sister-clade to LASV lineage II, which circulates in *Mastomys* populations within southwestern Nigeria, hinting the virus may have switched at some point between these two rodent species. Still, another remarkable feature of LASV circulation regarding central Benin is that, for the first time, different rodent-borne lineages of the virus have been found to co-occur in the same locality, Worogui. This demonstrates the consequential role of central Benin in the evolutionary history of LASV and its potential (up till now considerably underestimated) for even further emergence.

#### Previous infection

*M.* (*Nannomys*) *baoulei* and *L. striatus*, rodent species in which we detected LASV RNA within central Benin, also had individuals with IgG antibodies, indicating cleared infection. We also found antibodies in taxa that did not carry an active infection. IgG was present in one *P. daltoni* in Kadjola, central Benin; one *M. erythroleucus* from Lafia, central Nigeria; and four *M. erythroleucus* from Ndubia, south eastern Nigeria. (IgG signals without active infection in *M. erythroleucus* within Ndubia had similarly been found during 2015, prior to this survey^15^). Furthermore, IgG antibodies were detected in this present study in four *Mus musculus* and one *Crocidura* sp. from Jos (Fig. 3). Since antibodies to LASV are not exclusive and cross-react with other mammarenaviruses^15,42,51^, interpretation of IgG signals in small mammals can be subjective; especially when actively-infected conspecifics, from which an inference might be drawn, are not present in the vicinity. Several *P. daltoni* individuals have been found to be IgG-positive around LASV-infected *Mastomys* within Nigeria^15,16^. In spite of this, live infection has never been detected in *P. daltoni* and this rodent has not been confirmed as a natural LASV host. It is believed the serological signals in *P. daltoni* are an indication of transient spill-overs arising from proximity to *Mastomys* reservoirs within Nigeria; and in this present study, from reservoirs such as *M.* (*Nannomys*) *baoulei* and *L*. *striatus* within central Benin.

*M. erythroleucus* has been confirmed as a LASV reservoir within the countries of Guinea, Nigeria and Sierra Leone. We are inclined to interpret the *M. erythroleucus*-IgG detected in this study as LASV-induced because, hitherto, no live mammarenavirus infection other than LASV has been detected in this multimammate mouse within Nigeria; either within the Guinea savanna or the forest region^20^. On the other hand, we are reluctant to ascribe the IgG signals in *M. musculus* and *Crocidura* sp. to LASV, as, up to now, the live virus has never been detected in these species. In the face of widening mammarenavirus diversity being described across Africa, e.g.^57–60^, a range of candidates are possible. *M. musculus*, for instance, is the natural reservoir of *Mammarenavirus choriomeningitidis* (lymphocytic choriomeningitis virus—LCMV), the virus that causes Lymphocytic Choriomeningitis in humans. This ailment ranges in severity from flu-like symptoms to meningitis and encephalitis^61^ with infections reported in the Americas, Europe, Australia, and Japan^62^. The House mouse is an invasive rodent and has been commonly recorded along the coastline within Nigeria^31,47^. Encountering *M. musculus* in this study in Jos, central Nigeria, was surprising but reflects a recent pattern in other countries such as Senegal^63^ and Niger^46^ where this rodent is making human-assisted incursions into the West African interior, bearing the risk of LCMV. High topography (1217 m above sea level) favours a cooler climate in Jos that is more “temperate” in comparison to most other parts of Nigeria, with temperatures averaging 21 °C^35^. This, through colonial times till date, has attracted a flow of international visitors for tourism, missionary and mining purposes^64^, probably facilitating introduction of the House mouse. Our detection of the IgG-bearing *M. musculus* within the Guinea savanna of Nigeria further highlights how human-driven alteration of the small mammal community increases the risk of zoonoses caused by mammarenaviruses.

## Conclusion

LASV ecological studies are usually focussed on endemic countries, i.e., Nigeria and the Mano River Union region. Still, there are areas outside habitually recognised LASV hotspots where outbreaks occur among humans yet more insight is needed into ecology of the virus; such as the Guinea savanna belt stretching across Benin and Nigeria. Our small mammal survey showed that, along this savanna belt, prevalence of active LASV infections and co-occurrence between *M. natalensis* and *M. erythroleucus* (reservoirs known to be predominant in endemic areas) were limited. On the other hand, within central Benin in particular, non-*Mastomys* rodents such as *M.* (*Nannomys*) *baoulei* and *L. striatus* maintain an enclave of ongoing LASV infections—unnoticed until recently—that plays a significant role in the evolution and emergence of the virus. Further surveillance should be targeted at areas not currently regarded as endemic.

## Supplementary Information

Below is the link to the electronic supplementary material.

Supplementary Material 1

Supplementary Material 2

Supplementary Material 3

## Data Availability

Genetic sequences generated in this study (Lassa virus GP 1 kb, PX994346-48 and Cytochrome b, Lemniscomys striatus, PX994349-51) have been submitted to GenBank. The actual sequences are presented in the Supplementary files.
