# Supplementary material for: Non-Mastomys rodents harbour ancient Lassa virus lineages within Benin and Nigeria’s Guinea savanna belt
Source: Sci Rep. 2026 May 9;16:21306. doi: 10.1038/s41598-026-51525-8 (PMC13347048; doi:10.1038/s41598-026-51525-8)
Supplement: Supplementary file 3 — Supplementary Material 3 [file 41598_2026_51525_MOESM3_ESM.docx]

**Title: Non-*Mastomys* Rodents Harbour Ancient Lassa Virus Lineages within Benin and Nigeria’s Guinea Savanna Belt**

**Supplementary information**

**Authors**

Ayodeji Olayemi^1,2,*^, Adetunji Samuel Adesina^3^, Akinlabi Oyeyiola^1^, Adeoba Obadare^1^, Umaru Bangura^2^, Nnennaya Anthony Ajayi^4^, Kingsley Ukwaja^4^, Liman Mohammed^5^, Adamu Ibrahim^5^, Toni Rieger^6^, Stephan Günther^6^, Anges Yadouleton^7^ Elisabeth Fichet-Calvet^2^

**Affiliations**

1. Natural History Museum, Obafemi Awolowo University, Ile Ife, Osun State, Nigeria
2. Zoonoses Control Research Group, Bernhard Nocht Institute for Tropical Medicine, Hamburg, Germany
3. Department of Biochemistry and Molecular Biology, Obafemi Awolowo University, Ile Ife, Osun State, Nigeria
4. Federal Teaching Hospital Abakaliki, Abakaliki, Ebonyi State, Nigeria
5. Ministry of Health, Nasarawa State, Nigeria
6. Virology Department, Bernhard Nocht Institute for Tropical Medicine, Hamburg, Germany
7. Laboratoire des Fièvres Hémorragiques Virales, Cotonou, Benin

*Corresponding author

| **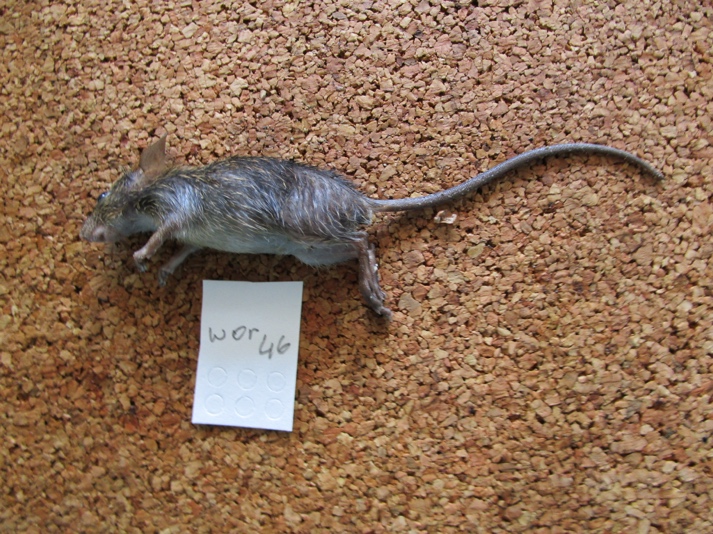** | **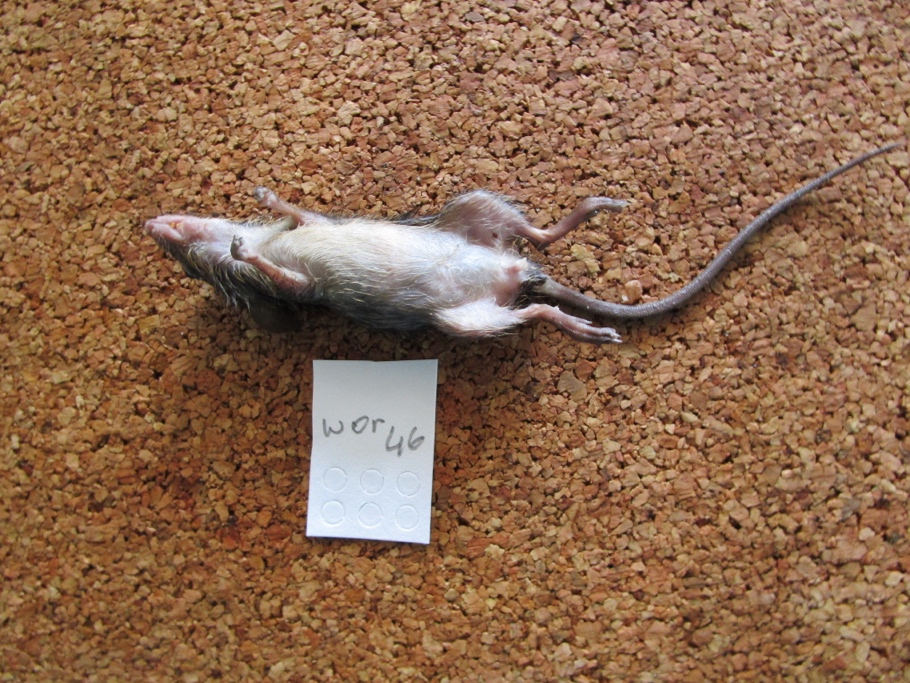** |
| --- | --- |
| ***Lemniscomys striatus* Worogui 46** |  |

| **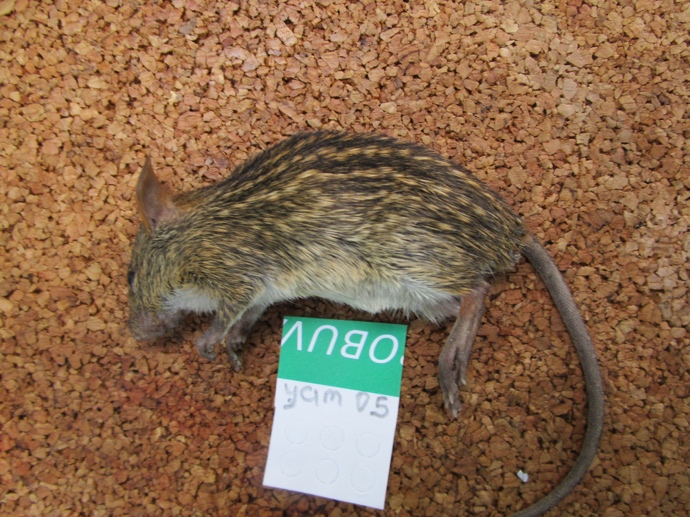** | **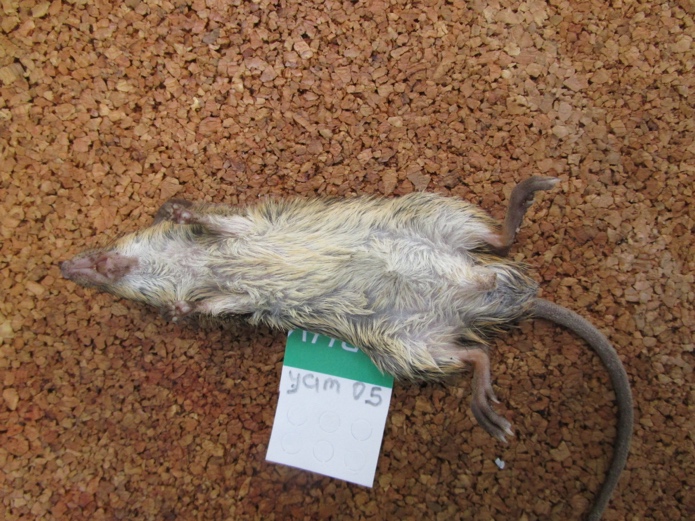** |
| --- | --- |
| ***Lemniscomys striatus* Yambouan 05** |  |

| **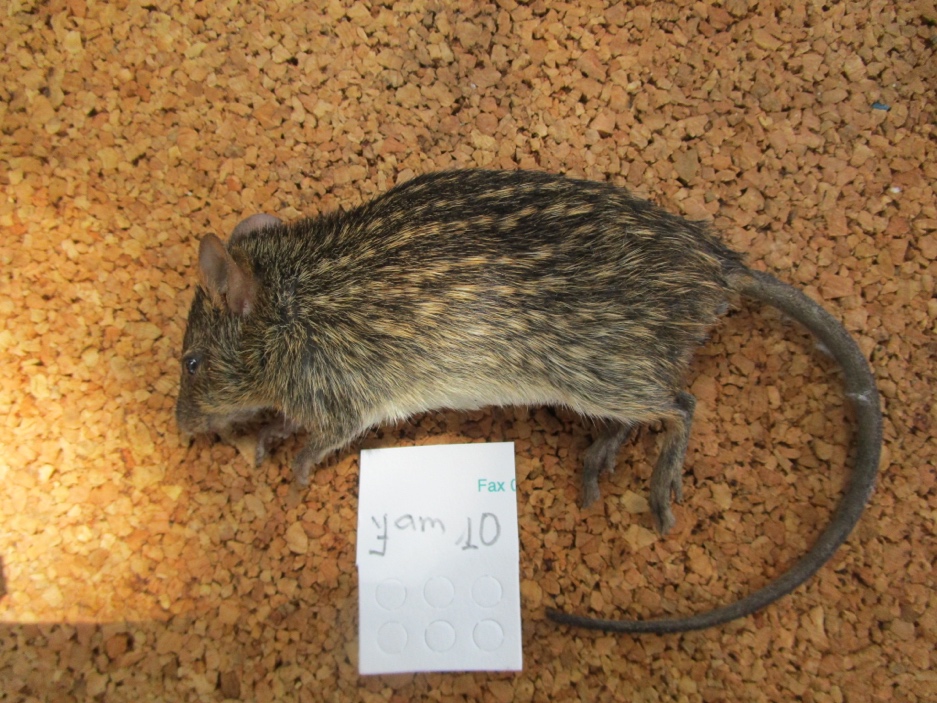** | **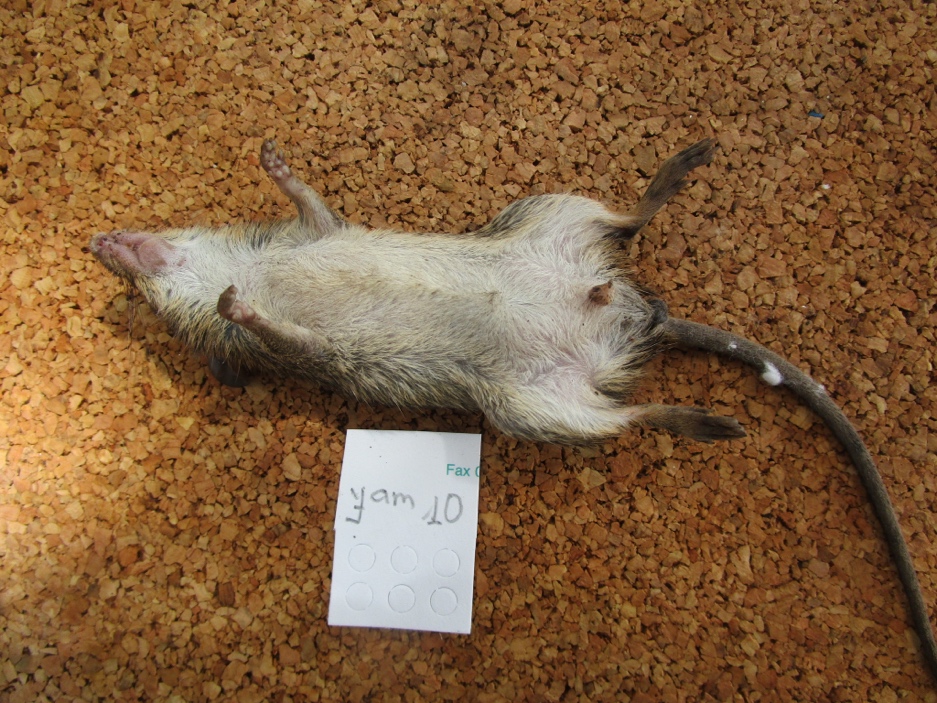** |
| --- | --- |
| ***Lemniscomys striatus* Yambouan 10** |  |

**Supplementary Figure S1:** Photos of the three LASV positive *Lemniscomys striatus* showing side and belly views. GenBank accession numbers for cytochrome b: PX994349-351.
